# A Japanese Remedy for Sterility

**Published:** 1851-03

**Authors:** E. Williams


*A Japanese Remedy for Sterility.* By Dr. E. Williams. —[Dr. Williams believes there is a botanical remedy in Japan and China which exerts a specific influence upon the uterus, more particularly in cases of sterility and checked menstruation. The tree is one of the order Ternstromaceæ of Jussieu, growing to the size of the English laurel, with leaves somewhat larger than the congou tea, and the odor resembling pulegium and sabina.]

The mode of preparation is to take a quantity of the leaves, macerate them in as much rice-spirit (samshu) as will just moisten them for six hours; then express and give about a teaspoonful every hour, and two or three doses will invariably bring on the menstrual secretion, which can be maintained by a dose or two daily for any length of time.

Females in the healthy state are expected to menstruate in their eighth year in Japan and China, and should they not do so they are inelligible for betrothal; therefore recourse is had to the *key tu sing* with certain results.

To ensure its success, according to popular belief, the leaves must be gathered by a virgin, the while using certain cabalistic formulæ, at the full of the moon, and during the burning of a certain number of highly perfumed jop-sticks.

When required for the purpose of obviating sterility, the tree must be in its second year, and also be gathered with certain prescribed formulæ at the wane of the moon, and equal parts of the root must be added to the preparation, which is made in the same manner as the preceding receipt.

That the root is aphrodisiac in its effects I have not the slightest doubt, as I have administered it to animals with obvious results, and without any ill effects even to mules and castrati.—*Lancet.*

